# Phospholipid dynamics in ex vivo lung cancer and normal lung explants

**DOI:** 10.1038/s12276-020-00547-x

**Published:** 2021-01-06

**Authors:** Julia Lesko, Alexander Triebl, Elvira Stacher-Priehse, Nicole Fink-Neuböck, Jörg Lindenmann, Freyja-Maria Smolle-Jüttner, Harald C. Köfeler, Andelko Hrzenjak, Horst Olschewski, Katharina Leithner

**Affiliations:** 1 Department of Internal Medicine, Division of Pulmonology, Medical University of Graz, 8036 Graz, Austria; 2 Core Facility Mass Spectrometry and Lipidomics, ZMF, Medical University of Graz, Graz, Austria; 3 Institute of Pathology, Medical University of Graz, Graz, Austria; 4 Division of Thoracic and Hyperbaric Surgery, Medical University of Graz, Graz, Austria; 5 Ludwig Boltzmann Institute for Lung Vascular Research, Graz, Austria; 6 Present Address: Institute of Pathology, Asklepios Clinic Munich-Gauting, Munich, Germany

**Keywords:** Cancer metabolism, Lung cancer

## Abstract

In cancer cells, metabolic pathways are reprogrammed to promote cell proliferation and growth. While the rewiring of central biosynthetic pathways is being extensively studied, the dynamics of phospholipids in cancer cells are still poorly understood. In our study, we sought to evaluate de novo biosynthesis of glycerophospholipids (GPLs) in ex vivo lung cancer explants and corresponding normal lung tissue from six patients by utilizing a stable isotopic labeling approach. Incorporation of fully ^13^C-labeled glucose into the backbone of phosphatidylethanolamine (PE), phosphatidylcholine (PC), and phosphatidylinositol (PI) was analyzed by liquid chromatography/mass spectrometry. Lung cancer tissue showed significantly elevated isotopic enrichment within the glycerol backbone of PE, normalized to its incorporation into PI, compared to that in normal lung tissue; however, the size of the PE pool normalized to the size of the PI pool was smaller in tumor tissue. These findings indicate enhanced PE turnover in lung cancer tissue. Elevated biosynthesis of PE in lung cancer tissue was supported by enhanced expression of the PE biosynthesis genes *ETNK2* and *EPT1* and decreased expression of the PC and PI biosynthesis genes *CHPT1* and *CDS2*, respectively, in different subtypes of lung cancer in publicly available datasets. Our study demonstrates that incorporation of glucose-derived carbons into the glycerol backbone of GPLs can be monitored to study phospholipid dynamics in tumor explants and shows that PE turnover is elevated in lung cancer tissue compared to normal lung tissue.

## Lung cancer: Identifying changes in lipid metabolism

Lung cancer cells rewire their metabolism to produce more of a lipid, or fat, called phosphatidylethanolamine (PE), an important component of the cell membrane. The ways that cancer cells change their metabolism to support tumor growth have been studied, but how lipid metabolism is altered is not well understood. Katharina Leithner at the Medical University of Graz in Austria and co-workers fed healthy lung tissue explants (fragments) and lung cancer explants with isotopically labelled sugars, and traced the metabolic products of these sugars. They found that lung cancer tissue made more PE than healthy tissue, and that the genes for PE biosynthesis were highly activated. These results demonstrate a new method for studying how cancer cells rewire lipid metabolism, and may help reveal cancer cells’ metabolic vulnerabilities, opening the way to development of novel treatments.

## Introduction

Lipogenesis, the de novo synthesis of lipids, is enhanced in many cancer types. Robust upregulation of fatty acid synthase (FASN) and other fatty acid biosynthetic genes has been noted in different cancers (for a review, see ref. ^1^). However, fatty acid import may also be upregulated in cancer cells and contribute to tumor growth in a manner dependent on the mutational landscape and context^2^. The bulk of fatty acids produced in cancer cells is predominantly used to generate glycerophospholipids (GPLs), the major subtype of membrane phospholipids^1,2^. GPLs are important structural and functional components of biomembranes and play an important role in cell signaling and organelle function^3^. They are structurally composed of a glycerol backbone linked to two fatty acid chains and different head groups, which define the GPL subclasses, for example, phosphatidylcholine (PC), phosphatidylethanolamine (PE), and phosphatidylinositol (PI). GPL synthesis proceeds mostly via lysophosphatidic acid (LPA), which is formed from glycerol-3-phosphate by glycerol-3-phosphate acyltransferases. LPA is then converted to phosphatidic acid (PA) by the addition of a second fatty acid chain. PA is dephosphorylated to generate diacylglycerol (DG), a precursor of PC or PE biosynthesis. Alternatively, PA is modified to produce cytidine diphosphate (CDP)-DG, a precursor of PI or cardiolipin biosynthesis^3^. In not only cancer cells but also normal proliferating cells, GPLs are continuously synthesized de novo, and their biosynthesis is balanced by their degradation^4,5^.

Levels of the PC and PE precursors phosphocholine and phosphoethanolamine have been shown to be increased in different cancers compared to the corresponding nonmalignant tissues^6^. In the past decade, progress in the knowledge of the phospholipidome under normal or pathological conditions has been fostered by the development of novel mass spectrometry techniques and analytical platforms, including electrospray ionization mass spectrometry approaches^7–9^. Intriguingly, comparative lipidomics showed marked differences in the composition of GPL in cancer tissues and the corresponding normal tissues (for reviews, see refs. ^7,8^). In non-small cell lung cancer (NSCLC), GPL profiles were shown to clearly discriminate between tumor and normal lung tissue^10,11^. A mouse model of mutant epidermal growth factor receptor-driven lung cancer recently showed complex alterations in lipid profiles, including decreases in different PI species and a saturated PE species (PE 16:0), while the contents of certain triglycerides and phosphatidylmethanol species were increased compared to those in normal lung tissue^12^. While GPL profiling appears to be a highly useful tool for discriminating between normal and cancer tissue, even in intraoperative settings^7,8^, the contribution of GPL synthesis and degradation to cancer biology remains incompletely understood. Lipin-1, one of the enzymes responsible for the conversion of PA to DG, was shown to be upregulated in basal-like triple-negative breast cancer, and silencing lipin-1 significantly inhibited tumor growth in a breast cancer model in vivo^13^. Lipin-1 expression was shown to be elevated in NSCLC compared to normal lung tissue, and silencing lipin-1 in NSCLC cells induced endoplasmic reticulum stress and reduced proliferation and survival^14^. While genetic manipulation of GPL biosynthetic genes provides valuable insights into their contributions to tumor growth, stable isotope tracing is needed to study the biosynthesis and degradation of different GPL classes in tumor tissues^15^. Here, we studied the relative dynamics of different GPL classes using ^13^C-labeled glucose in human NSCLC-derived explants.

## Materials and methods

### Lung cancer explants

Samples of tumor tissue and corresponding noninvolved normal lung tissue from six consecutive patients with NSCLC who were referred for surgical resection to the Division of Thoracic and Hyperbaric Surgery, Medical University of Graz, were included in the study Patients treated with preoperative chemotherapy were excluded from the study. Patient- and tumor-specific data are listed in Table 1. Surgical specimens were cut into small fragments using a razor blade immediately after surgery, and the fragments (explants) were incubated in 6-well plates (up to six fragments per well) in DMEM (Dulbecco’s modified Eagle’s medium; Gibco, Waltham, MA) supplemented with the different concentrations of labeled or unlabeled glucose and serum described below for up to 72 h and were then subjected to liquid chromatography with tandem mass spectrometry (LC-MS/MS) analysis, viability analysis or fixation and paraffin embedding, as described below. The mean duration between surgical removal and the start of explant culture was 2.5 h. Pathological diagnoses and the presence of tumor cells in the explants were confirmed by a pathologist (E.S.-P.). One patient had to be excluded since the tumor explants lacked tumor cells despite the initial diagnosis. The longest and shortest diameters of the tumor and lung explants from three patients were measured at the end of the experiments using an inverted microscope (Olympus Basic IX51, Olympus, Shinjuku, Tokyo, Japan). The size of the tumor explants (median largest diameter, 1.25 mm; range, 0.75–2.0 mm) was slightly smaller than that of the lung explants (median largest diameter, 1.82 mm; range, 1.0–2.6 mm). The study protocol was approved by the ethics review board of the Medical University of Graz. Signed informed consent was obtained from all patients prior to surgery.

**Table 1:** Patients’ clinical data.

Patient	#1	#2	#3	#4	#5	#6
Gender	M	F	F	F	M	F
Age at surgery (years)	57	74	58	75	54	83
Histology	LUAD	LUAD	LUAD	LUAD	LCNEM	LUAD
Postop TNM	pT3 N1 M0	pT2a N0 M0	pT3 pN1 M0	pT2b N0 M0	pT2a N0 M0	pT2a N0 M0
Stage	IIIA	IB	IIIA	IIA	IB	IB
Grade	G3	G2	G2	G2	G3	G2
Explant culture	non-st	non-st	non-st	st	st	non-st; st

*LUAD* lung adenocarcinoma, *LCNEM* large-cell carcinoma with neuroendocrine morphology, *postop TNM* post-operative TNM classification (7th edition), *non-st* nonstarvation, *st* starvation.

### Stable isotope labeling

After removal and dissection, tumors and lung explants were cultured in DMEM (Gibco, Waltham, MA) supplemented with different concentrations of labeled or unlabeled glucose, 1 mM glutamine (Sigma-Aldrich, St. Louis, MO, USA), 100 U/ml penicillin and 100 µg/ml streptomycin (Gibco), with or without dialyzed fetal calf serum (FCS). For culture under “nonstarvation” conditions, media were supplemented with 5 mM glucose (Sigma) or [^13^C_6_]glucose (Cambridge Isotope Laboratories, Tewksbury, MA, USA) and 10% dialyzed FCS (Gibco). For culture under “starvation” conditions, media were supplemented with 1 mM glucose (Sigma) or [^13^C_6_]glucose (Cambridge Isotope Laboratories) without FCS. Media were replaced every 24 h.

### Sample preparation for MS

Lipid extraction was performed as described^16^ with minor modifications. Explants were briefly washed with glucose- and glutamine-free medium and were then transferred to 1.5 ml of ice-cold methanol and homogenized with an Ultra-Turrax tissue homogenizer prior to three pulses of sonication for 10 s. PC 24:0 (5 nmol) was added as an extraction control. After the addition of 2.5 ml of methyl-*tert*-butyl ether (MTBE), the mixture was vortexed for 10 s and sonicated for 10 min. After further addition of 2.5 ml of MTBE and 10 min of shaking, 1.25 ml of deionized water was added, and the mixture was subjected to 10 min of overhead shaking. Thereafter, the samples were centrifuged (3 min at 1350 × *g*, 21 °C), and the upper organic phase was stored. The lower aqueous phase was reextracted with 2 ml of the upper phase of MTBE/methanol/water (10/3/2.5, v/v/v). The resulting upper phases were combined and evaporated in a vacuum centrifuge. The dried lipids were resuspended in 1 ml of chloroform/methanol (1/1, v/v).

### Hydrophilic interaction liquid chromatography/MS (HILIC/MS) for analysis of isotopic enrichment of GPL

Chromatography was performed as described^16^. Briefly, the separation was performed on a Phenomenex Kinetex HILIC column (2.1 mm × 100 mm, 2.6 µm) (Phenomenex, Aschaffenburg, Germany) thermostatted to 50 °C in a Thermo Accela 1250 HPLC System (Thermo Fisher Scientific, Waltham, MA). Mobile phase A comprised deionized water containing 1% (v/v) aqueous ammonium formate (1 M) and 0.1% (v/v) formic acid. Acetonitrile/isopropanol (5/2, v/v) with the same additives was used as mobile phase B. Gradient elution started at 95% mobile phase B and was decreased to 75% mobile phase B over 10 min. The column was reequilibrated for 8 min with 95% B. The flow rate was set to 250 µl/min. Two microliters of sample, thermostatted to 10 °C, was injected using full loop injection mode.

An Orbitrap Velos Pro mass spectrometer (Thermo Fisher Scientific) was operated in negative ion mode alternating between full MS and tandem MS modes. Full scan spectra were acquired at a resolution of 30,000. Tandem mass spectra were acquired at a resolution of 7500. Phospholipid classes were analyzed using higher-energy collisional dissociation activation (normalized collision energy, 75; activation time, 0.1 ms; FT first mass mode, fixed at mass-to-charge (*m/z*) 100). For the first 3 min, PI molecular species were isolated (*m/z* 887.5 ± 75); for the next 3 min, PE molecular species (*m/z* 745.5 ± 75); and for the remainder of the gradient, PC molecular species (*m/z* 804.5 ± 100). Xcalibur Quan Browser and Microsoft Excel were used for data processing. For each lipid class investigated (PI, PE, and PC), two sets of fragment ions were analyzed: one set of head group fragment ions containing glycerol and one set of fragments not containing glycerol. For each set, the extracted ion chromatograms of five isotopologues (containing zero, one, two, three, or four ^13^C atoms) were integrated using Lipid Data Analyzer^17^. The chemical structures of the target fragments have been reported in ref. ^16^. The exact *m/z* values are as follows: PC containing glycerol, 224.07; PC not containing glycerol, 168.04; PE containing glycerol, 196.04; PE not containing glycerol, 140.01; PI containing glycerol, 297.04; and PI not containing glycerol, 241.01. The areas of the isotopologues were normalized to the area of the corresponding monoisotopic ion. The isotopologue abundance was corrected for the natural abundance of ^13^C using IsoCor software^18^. The normalized total pool sizes were determined by calculating the ratio of the PE area to the PI or PC area.

### MTS assay

An MTS Cell Proliferation Colorimetric Assay Kit (Biovision, Milpitas, CA, USA) was used to assess explant viability according to the manufacturer’s instructions. This method is based on the reduction of MTS tetrazolium caused by viable cells to yield a soluble, colored formazan product. Briefly, 20 µl of MTS reagent (Biovision) was added to phenol red-free culture medium supplemented with 10% dialyzed fetal bovine serum (Gibco) and antibiotics (Gibco). Fragments were incubated in MTS-containing medium for 3 h in 96-well plates. Then, the fragments were washed in PBS, patted gently to remove excess the medium, and weighed separately. The optical density (OD) of the medium was analyzed in a spectrophotometer (SPECTRAmax PLUS 384, Molecular Devices, Sunnyvale, CA, USA) and normalized to the weight of each fragment. Tumor and lung fragments treated with 70% ethanol for 30 min were used as a negative control.

### Gene expression analysis using publicly available datasets

Gene expression data of GPL biosynthetic genes were retrieved from the TCGA (The Cancer Genome Atlas) lung adenocarcinoma (LUAD) and lung squamous cell carcinoma (LUSC) datasets using the UCSC Xena platform (https://xenabrowser.net/). TCGA gene expression data were generated by the TCGA Research Network (https://www.cancer.gov/tcga).

## Results

### Determination of lung and tumor explant viability

**Fig. 1:**
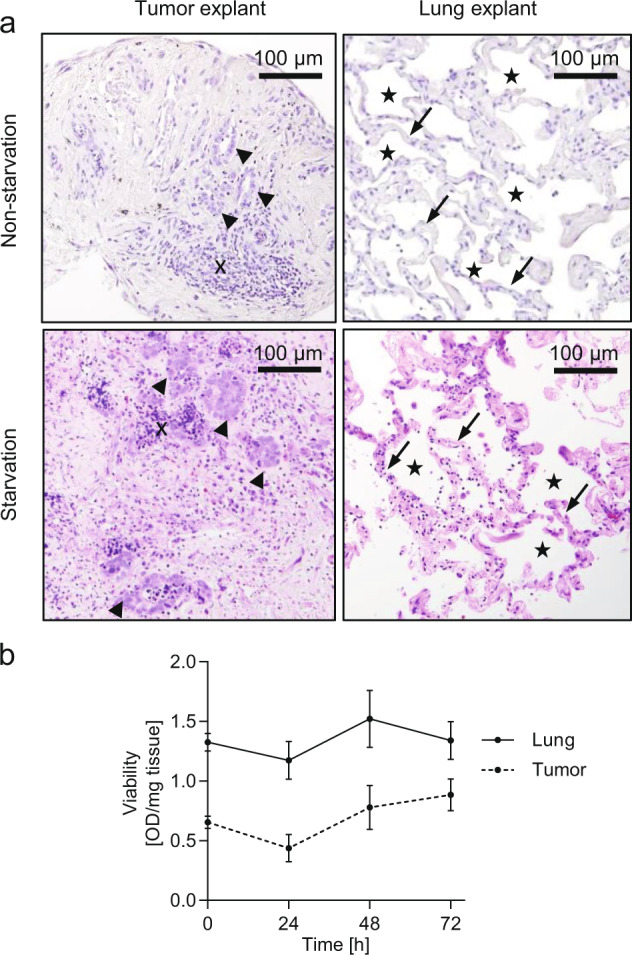
Morphological appearance and viability of lung and tumor explants. **a** Hematoxylin and eosin staining of the tumor and the corresponding normal lung explants after 72 h of culture. An example of lung adenocarcinoma explants cultured in nonstarvation medium containing 5 mM glucose and 10% dialyzed FCS (upper row) and an example of a large-cell neuroendocrine carcinoma cultured in starvation medium containing 1 mM glucose without FCS (lower row) are shown. Arrowheads, tumor cell nests; x, lymphocytes; arrows, alveolar walls; stars, alveolar spaces. **b** Viability of normal lung and tumor explants cultured under starvation conditions for different time intervals. Weight-corrected OD values are shown as the mean ± SEM values. At least *n* = 10 explants from a total of three patients were analyzed at each time point. No significant difference between viability immediately after dissection (0 h) and at later timepoints was found.

In solid tumors, blood perfusion is frequently inadequate, and steep local gradients for glucose and other nutrients are created^19^. On the other hand, the availability of nutrients is an important determinant of cancer cell metabolism^20,21^. Thus, we aimed to study cancer metabolism in human NSCLC explants under conditions of normal fasting blood levels of glucose (5 mM) in the presence of serum (“nonstarvation conditions”) or under conditions of 1 mM glucose in the absence of serum (“starvation conditions”). First, we assessed the morphology of the lung and NSCLC explants cultured in the respective media. The architecture of the freshly isolated tissue and the cultured explants was similar, although signs of decreased viability were noted after 72 h of culture (Fig. 1a). Lung fragments showed moderate destruction of the alveolar structure and focally slightly thickened septa. Tumor fragments exhibited variable degrees of necrotic changes, which were more pronounced under starvation, but no explant was entirely necrotic (Fig. 1a). Tumor explants contained separated tumor cells and tumor cell nests and a large number of stromal cells and some lymphocytes. To assess lung and tumor explant viability under starvation conditions, we performed MTS assays. The weight-corrected OD values at 24, 48, and 72 h of culture were not significantly different from the value at 0 h (Fig. 1b).

### Contribution of de novo GPL synthesis to GPL pools

**Fig. 2:**
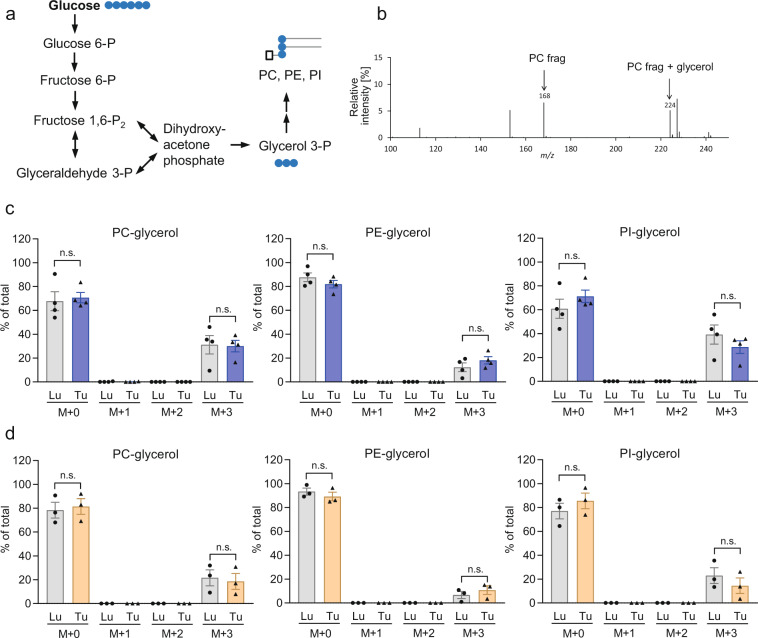
Abundances of GPL-glycerol isotopologues in lung and tumor explants. **a** Metabolic pathway for the generation of phospholipid glycerol backbone from glucose. **b** Mass spectrum showing signal intensities at different mass-to-charge (*m/z*) ratios. The phosphatidylcholine (PC) head group fragment ion not containing glycerol (PC frag) and the corresponding fragment containing glycerol (PC frag + glycerol) are highlighted. **c**, **d** After 72 h of culture with [^13^C_6_]glucose under either nonstarvation conditions (**c**) or starvation conditions (**d**), explants were analyzed by LC-MS/MS, and the isotopologue abundances of GPLs containing labeled or unlabeled glycerol moieties was determined. M + 0 denotes unlabeled glycerol-containing fragments, while M + 3 denotes fully ^13^C-labeled glycerol. No M + 1 or M + 2 (partially labeled glycerol) fragments were found. Natural abundance-corrected data from four (**c**) or three (**d**) patients are shown as the mean ± SEM values. PC phosphatidylcholine, PE phosphatidylethanolamine, PI phosphatidylinositol, Lu lung, Tu tumor, n.s. nonsignificant.

We utilized fully ^13^C-labeled glucose under starvation and nonstarvation conditions to assess the contribution of de novo GPL synthesis to the total GPL abundance in lung and NSCLC explants. The duration of labeling was 72 h, and the medium was changed daily. The labeled glucose was metabolized to glycerol-3-phosphate, which is required for the formation of the glycerol backbone of GPL (scheme depicted in Fig. 2a). Fragments of GPL backbones either containing or not containing glycerol were measured, and the relative isotopologue abundance values were calculated. Figure 2b shows a mass spectrum of the different PC fragments. All three GPL classes analyzed, PC, PE, and PI, showed incorporation of the ^13^C label into the glycerol backbone in the lung as well as NSCLC tissue (Fig. 2c, d). PC and PI showed the highest isotopologue enrichment values of up to 40% in the glycerol moiety (i.e., 40% of the PC and PI pools were derived from de novo biosynthesis by acylation of fatty acids to GPL precursors containing labeled glycerol). As expected, the glycerol-containing fragments were either unlabeled or contained an M + 3 label, corresponding to fully ^13^C-labeled glycerol (Fig. 2c, d). No label transfer from glucose to GPL fragments containing GPL head groups but not glycerol was found (Supplementary Fig. S1). Glucose is also a precursor for acetyl-CoA, which is required for fatty acid biosynthesis. However, fatty acids may also be imported from (serum-containing) medium or recycled from preexisting phospholipids for de novo GPL biosynthesis. Thus, we did not assess label transfer to the fatty acid moieties of GPL to determine GPL turnover.

### Enhanced contribution of de novo PE biosynthesis to the total PE pool in tumors

**Fig. 3:**
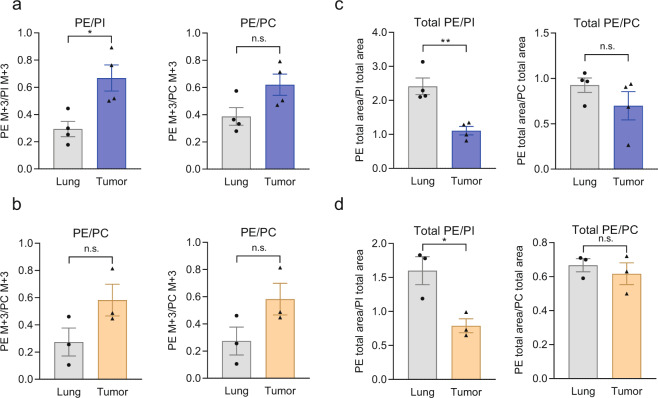
Contribution of de novo synthesis to GPL pools during ex vivo culture of lung and tumor explants. After 72 h of culture with [^13^C_6_]glucose under either nonstarvation conditions (**a**) or starvation conditions (**b**), explants were analyzed by LC-MS/MS, and the isotopologue abundance of PE containing ^13^C-labeled glycerol moieties was determined and normalized to the abundance of PI or PC to account for possible differences in precursor enrichment. M + 0 denotes unlabeled glycerol-containing fragments, while M + 3 denotes fully ^13^C-labeled glycerol. Relative abundances of PE, PI, and PC. **c**, **d** Relative total abundance (labeled and unlabeled isotopologues) of PE normalized to PI or PC in [^13^C_6_]glucose-treated cells cultured under nonstarvation (**c**) or starvation conditions (**d**). Data from four (**a**, **c**) or three (**b**, **d**) patients are shown as the mean ± SEM values. PC phosphatidylcholine, PE phosphatidylethanolamine, PI phosphatidylinositol. *P < 0.05 and **P < 0.01 by two-tailed Student’s *t* test; n.s., nonsignificant.

Unlabeled GPL-glycerol fragments were derived from either preexisting GPL not subjected to turnover or de novo synthesized GPL formed from unlabeled glycerol-3-phosphate. To account for possible differences in glucose uptake and glycerol-3-phosphate labeling in the different tissues, we normalized the abundance of the M + 3 PE-glycerol isotopologue to that of M + 3 PI-glycerol or M + 3 PC-glycerol. Under both experimental conditions, that is, nonstarvation and starvation, tumor explants displayed markedly elevated PE/PI enrichment ratios compared with those of normal lung explants (Fig. 3a, b). A similar but nonsignificant difference was found in the PE/PC ratios (Fig. 3a, b). The enhanced PE labeling was not affected by the level of glucose or serum in the labeling medium. Interestingly, the relative sizes of GPL pools were deregulated in the opposite direction in tumor tissue compared to normal lung tissue (Fig. 3c, d). In tumor tissue, the ratio of total PE to PI was significantly decreased under both experimental conditions (Fig. 3c, d). In summary, the larger contribution of de novo synthesis to the PE abundance in tumors and the smaller pool size suggests a relatively higher rate of PE turnover and a lower rate of PI turnover in lung cancer than in normal lung tissue.

### PE biosynthesis genes are upregulated in NSCLC compared to normal lung tissue and are associated with poor survival

**Fig. 4:**
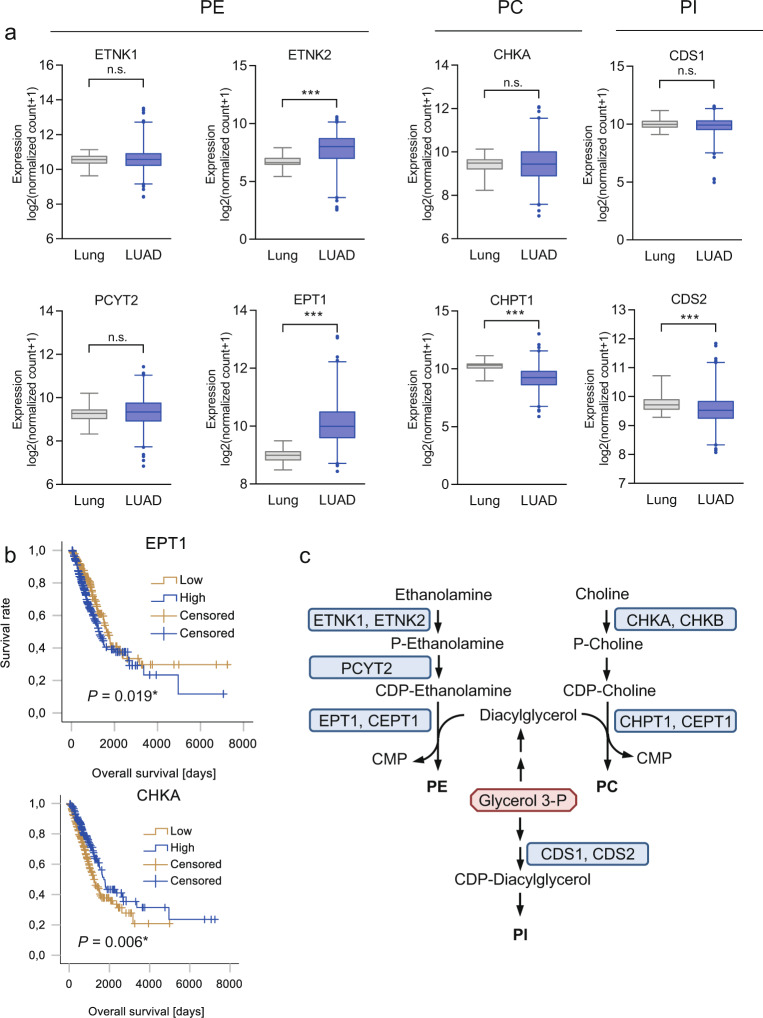
Expression levels of GPL biosynthesis genes in lung adenocarcinoma and noninvolved lung tissue and their association with outcomes. **a** Gene expression in lung adenocarcinoma (LUAD, *n* = 514) and noninvolved lung tissue (lung, *n* = 59) was obtained from the publicly available TCGA dataset. The genes were grouped according to their involvement in PE (phosphatidylethanolamine), PC (phosphatidylcholine), or PI (phosphatidylinositol) biosynthesis. Data were obtained via the XENA database (https://xenabrowser.net/). Group comparisons were performed by Mann–Whitney *U* tests. ***P < 0.001. n.s. Not significant. **b** Overall survival in patients with surgically treated lung adenocarcinoma showing high or low expression of the PE biosynthetic enzyme EPT1 or the PC biosynthetic enzyme CHKA (*n* = 491; data were retrieved via the UCSC Xena platform, https://xenabrowser.net/). Median values were used as the cutoffs. Differences in survival were assessed using the log-rank test. **c** Biosynthetic pathways for the different phospholipid classes.

PE can be synthesized in mammalian cells via the CDP-ethanolamine pathway, by base exchange pathways from phosphatidylserine (PS) or PC, or by acylation of lyso-PE. Only the CDP-ethanolamine pathway represents true de novo synthesis^22^. The biosynthesis of CDP-ethanolamine starts with phosphorylation of ethanolamine by ethanolamine kinase (ETNK1 or ETNK2; scheme depicted in Fig. 4c)^22^. Phosphoethanolamine is then converted to CDP-ethanolamine by ethanolamine-phosphate cytidylyltransferase (encoded by *PCYT2*)^22^. The final step, the transfer of phosphoethanolamine from CDP-ethanolamine to DG, is catalyzed either by choline/ethanolamine phosphotransferase 1 (CEPT1), which has dual activity toward both CDP-ethanolamine in the PE biosynthesis pathway and CDP-choline in the PC biosynthesis pathway, or by the recently discovered selenoenzyme ethanolamine phosphotransferase 1 (EPT1, also referred to as SELENOI)^22^. The main pathway for the biosynthesis of PC is the Kennedy (CDP-choline) pathway. In this pathway, choline is phosphorylated by choline kinase (CHKA or CHKB) to form phosphocholine. Phosphocholine is then metabolized to CDP-choline and finally transferred to DG by the choline phosphotransferases CHPT1 (also known as CPT1) and CEPT1, which is also responsible for PE biosynthesis^23^. Unlike PC and PE, PI is synthesized from CDP-DG^3^. CDP-DG synthase (CDS), with its two isoforms CDS1 and CDS2, activates PA to form the intermediate CDP-DG, which is then converted by PI synthase to PI. PA can be synthesized de novo or generated at the plasma membrane by the action of phospholipase C, which leads to hydrolysis of phosphatidylinositol (4,5) bisphosphate, producing DG. DG is then rapidly phosphorylated to PA^24^. CDS1 and CDS2 are also the starting points for the synthesis of other, less abundant GPLs, including cardiolipins^3^.

We analyzed the expression of PE, PC, and PI biosynthetic genes in publicly available TCGA datasets of LUAD and LUSC—the two main subtypes of NSCLC—and in normal lung tissue. *ETNK2*, an ethanolamine kinase, and *EPT1*, the enzyme mediating the final step of PE biosynthesis, were significantly upregulated in lung adenocarcinoma (Fig. 4a) and in squamous cell carcinoma (Supplementary Fig. S2). *ETNK1* was either not differentially expressed between tumor and lung tissue (adenocarcinoma) or showed slightly lower expression in tumor than in lung tissue (squamous cell carcinoma). *PCYT2*, responsible for activation of phosphoethanolamine, was significantly upregulated in squamous cell carcinoma (Supplementary Fig. S2). In contrast, the PC biosynthesis gene *CHPT1* was significantly downregulated in both NSCLC subtypes (Fig. 4a and Supplementary Fig. S2). Similarly, the PI biosynthetic genes *CDS1* and CDS2 were downregulated in squamous cell carcinoma, and *CDS2* was downregulated in adenocarcinoma compared to normal lung tissue (Fig. 4a, Supplementary Fig. S2). Importantly, ETNK2 and EPT1 exclusively mediate the biosynthesis of PE, in contrast to CHKB and CEPT1, which mediate the corresponding steps in PE biosynthesis but are also involved in PC biosynthesis (Fig. 4c). In lung adenocarcinoma, high expression of *EPT1* but low expression of *CHKA*, a choline kinase, was significantly associated with poor overall survival (Fig. 4b).

## Discussion

Cancer cells undergo metabolic reprogramming to support the biosynthesis of precursors for biomass production^25,26^. Identification of major biosynthetic pathways that are crucial for the maintenance of certain metabolite pools might reveal specific metabolic vulnerabilities of cancer cells^25,26^. To date, only a few studies have addressed the role of *de novo* GPL synthesis in lung cancer. In our study, we found an enhanced contribution of de novo GPL synthesis to PE pools in NSCLC compared to normal lung tissue. Moreover, we found enhanced expression of PE biosynthetic genes in different subtypes of NSCLC compared to normal lung tissue.

In this study, we sought to determine relative differences in GPL isotopic labeling in NSCLC and normal lung explants, focusing on three major GPL classes—PC, PE, and PI. The method used relies on labeling of the glycerol phosphate precursor in the cultured explants and on quantitative determination of the glycerol moiety in the different GPL classes by MS/MS using a previously established detection protocol^16^. A similar approach using stable isotope-labeled glucose was recently applied to label the glycerol phosphate precursor of the GPL backbone to infer changes in GPL dynamics^27^. The half-life of the glycerol moiety of membrane GPL was determined to be an average of 29 h in rats^28^, and it ranged from 2 to 20 days for different GPL classes in a *Drosophila* model^27^. The long half-lives of GPL reported in the literature, however, suggest that an isotopic steady state is not achieved during the 72-h labeling period. Under such non-steady-state labeling conditions, the increase in label enrichment is determined by the metabolic flux of label incorporation, on the one hand, and by the pool size, on the other hand^29^. In fact, the pool sizes were found to be inversely related to the fractional glycerol enrichment values (Fig. 3c, d). The observed faster incorporation of glycerol into PE (normalized to PI) in the tumors and the lower size of the PE pool (normalized to the PI pool) showed that the relative turnover of PE is higher in tumor than in normal lung tissue. Due to the inherent limitation in tissue availability, we did not perform enrichment measurements at different time points in the tumor and lung explants to accurately determine the half-lives of the different GPLs (i.e., increases in labeled GPLs and decreases in unlabeled GPLs).

PE is one of the most abundant classes of GPL in cell membranes^3^. Due to its conic shape, it promotes membrane curvature formation. However, PE also plays a major role in the regulation of other cellular functions, including autophagy^3^. The results of our study suggest that the expression of PE synthesis genes is upregulated and PE turnover is enhanced in NSCLC relative to the biosynthesis dynamics of other GPL classes. In a mouse model of KRAS-driven lung adenocarcinoma and GPL synthesis genes, as well as FA synthesis genes were among the top upregulated genes, indicating that lipogenesis is enhanced during KRAS-driven carcinogenesis^30^. PE synthesis genes were not included in the array used in that study. In a liver cancer model, inhibition of PE synthesis by meclizine in combination with glycolysis inhibition reduced tumor growth^31^. The results of the latter study suggest an interplay between PE synthesis and starvation in cancer. In fact, PE plays an important role in autophagy. Autophagy, a process of self-digestion, ensures the degradation of damaged organelles in not only normal but also cancer cells and provides metabolites under starvation conditions^32^. Covalent binding of PE to LC3, a key autophagy mediator, is required for its activity^32^. De novo PE synthesis was recently shown to proceed despite a lack of glucose in starved lung cancer cells by activation of gluconeogenesis, which provides glycerol phosphate from noncarbohydrate precursors^16^. Thus, PE might be required in cancer cells, especially under conditions of nutrient limitation and autophagy. In our study, we did not find major differences in PE-glycerol tracing in media containing 5 mM ^13^C-glucose and 1 mM ^13^C-glucose. However, our results were obtained in NSCLC explants from different patients, since too little tumor material was available from some patients to study all experimental conditions. The impact of nutritional status on de novo PE synthesis and PE turnover in cancer should thus be addressed in future studies. De novo synthesis of all three studied GPL classes, PE, PC, and PI, continued during explant culture in NSCLC as well as in normal lung explants. Stable isotope enrichment in the glycerol moiety was increased in PE relative to PI in lung cancer compared to normal lung tissue. Although a similar trend was noted in the PE/PC ratio, a contribution of decreased PI biosynthesis rates in lung cancer cells or altered PI pool sizes cannot be excluded. Interestingly, the expression of CDS1, a PI biosynthetic enzyme, has been shown to be reduced by ZEB1, an important mediator of epithelial-to-mesenchymal transition^33^. Interestingly, p53, an important tumor suppressor, has been shown to enhance the expression of CDS1 and CDS2^34^.

Cancer cells are at risk of undergoing ferroptosis, a mode of cell death induced by iron-dependent lipid peroxidation, partially due to an elevated intracellular iron content^35^. Polyunsaturated fatty acids are particularly prone to undergo peroxidation and trigger ferroptosis unless protected by glutathione peroxidase 4, a phospholipid peroxidase^36,37^. The predominant GPL responsible for ferroptosis induction is still controversial. Recently, PEs containing PUFAs have been suggested to drive ferroptosis^38,39^. Thus, enhanced PE biosynthesis in cancer cells might be required to balance PE damage due to peroxidation.

Despite these recently discovered links between PE biosynthesis and cell survival and homeostasis, the role of PE biosynthetic enzymes in cancer progression is unclear. Here, we found that the expression of several PE biosynthetic genes, including *ETNK2* and *EPT1*, was highly increased in tissues from two different subtypes of NSCLC compared to normal lung tissues, while the expression of PC and PI biosynthetic genes was decreased. Moreover, high expression of *EPT1* was associated with poor overall survival in adenocarcinoma. In a previous study, *ETNK1* was found to be amplified in a subset of lung adenocarcinomas, and *ETNK1* amplification was found to be correlated with poor prognosis^40^.

Cultured explants or slices derived from human tumors are increasingly being utilized as a tumor model that maintains cell diversity and tissue architecture (for reviews, see refs. ^41,42^). In cancer metabolism studies, this model allows the use of a defined and flexible tracer and the delineation of metabolic activity in the target tissue without systemic influences^20,41^. Not only stromal cells, such as fibroblasts and endothelial cells, but also immune cells present in human tumor tissue, are present in explants^43^. An advantage of this model is the ability to study tumor metabolism in its physiological 3D context. The observed changes in GPL dynamics occurred in the bulk of the tumor. Whether PE biosynthesis occurs primarily in neoplastic cancer cells or in stromal cells cannot be inferred from the data. In fact, cancer-associated fibroblasts from colon cancers have been shown to display enhanced expression of FASN and to release increased amounts of triglycerides and different GPLs, which are taken up by colon cancer cells^44^. Alterations in GPL dynamics in the different cell types within a tumor, for example, neoplastic tumor cells and stromal cells, should thus be assessed in future studies. In conclusion, this study shows that the incorporation of stable isotopic tracer-derived carbons into the glycerol backbone of GPL can be monitored in tumor explants, providing important insights into phospholipid dynamics in cancer. Although the number of patients was limited in this exploratory study, the data show that PE turnover and the expression of PE biosynthesis genes are strongly elevated in lung cancer tissue compared to normal lung tissue.

## Supplementary information

Supplementary File
