# Supplementary material for: Phospholipid dynamics in ex vivo lung cancer and normal lung explants
Source: Exp Mol Med. 2021 Jan 6;53(1):81–90. doi: 10.1038/s12276-020-00547-x (PMC8080582; doi:10.1038/s12276-020-00547-x)
Supplement: Supplementary file 1 — Supplementary File [file 12276_2020_547_MOESM1_ESM.pdf]

**Supplementary figure legends – Lesko et al.**

**Supplementary Fig. S1. Isotopologue abundance of PL fragments not containing glycerol in lung and tumor explants.** After 72 hours of culture in [$^{13}C_6$]glucose either under nonstarvation conditions (**a**) or starvation conditions (**b**), isotopologue abundance of PL fragments not containing glycerol was analyzed as a control. Natural abundance-corrected data from four (**a**) or three (**b**) patients are shown as mean +/- SEM. PC, phosphatidylcholine; PE, phosphatidylethanolamine; PI, phosphatidylinositol; Lu, lung; Tu, tumor; n.s. non-significant.

**Supplementary Fig. 2. Expression of PL biosynthesis genes in lung squamous cell carcinoma and non-involved lung tissue.** Gene expression in lung squamous cell carcinoma (LUSC, n = 501) and in non-involved lung tissue (Lung, n = 49) from the publically available LUSC TCGA dataset. Results are grouped according to the involvement of the enzymes in the biosynthesis of PE (phosphatidylethanolamine), PC (phosphatidylcholine), or PI (phosphatidylinositol). Data was obtained via the XENA database (https://xenabrowser.net). Group comparisons were performed by Mann-Whitney-U-tests. * $P < 0.05$; *** $P < 0.001$.

# Fig. S1

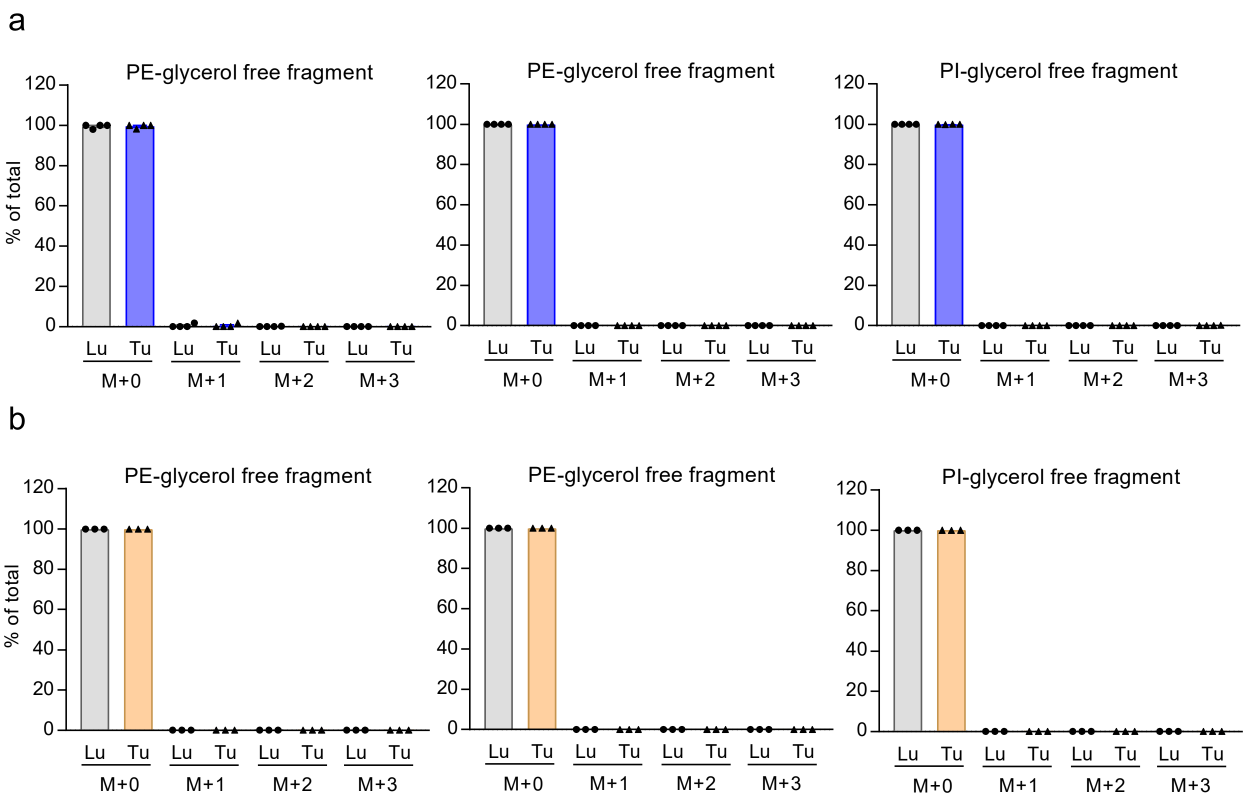
a
PE-glycerol free fragment
PE-glycerol free fragment
PI-glycerol free fragment
% of total
120
100
80
60
40
20
0
Lu Tu Lu Tu Lu Tu Lu Tu
M+0 M+1 M+2 M+3
b
PE-glycerol free fragment
PE-glycerol free fragment
PI-glycerol free fragment
% of total

# Fig. S2

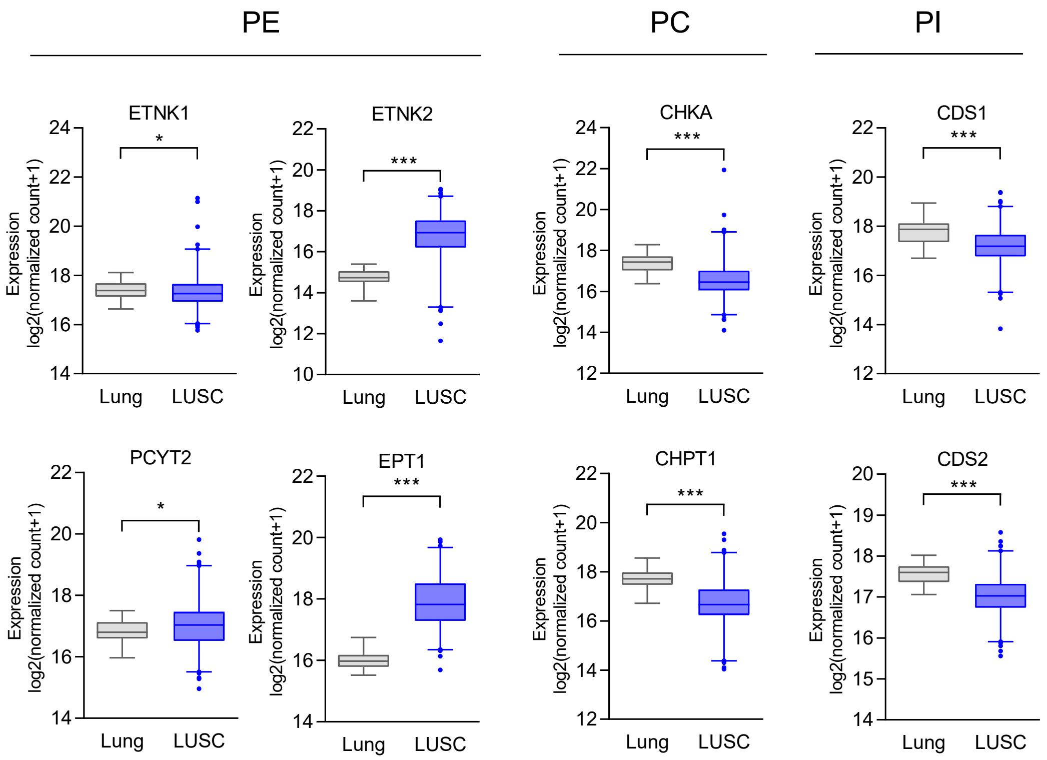
PE
PC
PI
ETNK1
ETNK2
CHKA
CDS1
PCYT2
EPT1
CHPT1
CDS2
Expression
log2(normalized count+1)
Lung
LUSC
*
***
